# Predictive value of acoustic cardiography for post-PCI early ventricular remodeling in acute myocardial infarction

**DOI:** 10.1038/s41598-023-34370-x

**Published:** 2023-05-03

**Authors:** Weiwei Wang, Haizhen Hao, Tingting Fan, Jia Yue, Mingyang Wang, Moshui Chen, Guolan Deng, Liangyi Si, Fuwei Zhang

**Affiliations:** 1 Division of Cardiology, Third Affiliated Hospital of Chongqing Medical University, Chongqing, China; 2 Department of Neurology, Haikou People’s Hospital, Central South University, Haikou, China; 3 Department of Critical Care Medicine, Peoples Hospital of Jiangbei District, Chongqing, China; 4 Division of Cardiology, The First Affiliated Hospital of Chongqing Medical University, Chongqing, China

**Keywords:** Cardiology, Cardiac device therapy

## Abstract

Acoustic cardiography is a completely new technology, it has great advantages in the rapid diagnosis of cardiovascular diseases. The purpose of this study was to investigate the clinical value of the fourth heart sound (S4), cardiac systolic dysfunction index (SDI), and the cardiac cycle time-corrected electromechanical activation time (EMATc) in the prediction of post-percutaneous coronary intervention (PCI) early ventricular remodeling (EVR) in patients with acute myocardial infarction (AMI). We recruited 161 patients with AMI of 72-h post-PCI, including 44 EVR patients with left ventricular ejection fraction (LVEF) < 50% and 117 Non-EVR patients (normal left ventricular systolic function group, LVEF ≥ 50%). EMATc, S4, and SDI were independent risk factors for post-PCI early ventricular remodeling in patients with AMI [S4 (*OR* 2.860, 95% *CI* 1.297–6.306, *p* = 0.009), SDI (*OR* 4.068, 95% *CI* 1.800–9.194, *p* = 0.001), and EMATc (*OR* 1.928, 95% *CI* 1.420–2.619, *p* < 0.001)]. The area under the receiver operating characteristic curve for EMATc was 0.89, with an optimal cutoff point of 12.2, EMATc had a sensitivity of 80% and a specificity of 83%. By contrast, an optimal cutoff point of 100 pg/ml, Serum brain natriuretic peptide had a sensitivity of 46% and a specificity of 83%. Our findings suggest the predictive value of EMATc for the occurrence of EVR in these patients was also identified; EMATc may be a simple, quick, and effective way to diagnose EVR after AMI.

## Introduction

Acute myocardial infarction (AMI) is a common cardiovascular disease that poses a serious threat to human health. Percutaneous coronary intervention (PCI) and standardized drug therapy can effectively treat most patients, but many patients still develop ventricular remodeling, which can contribute to heart failure post-PCI^1^. A strong relationship has been demonstrated among disease prognosis, heart failure, and ventricular remodeling after AMI. Ventricular remodeling is an independent predictor of the development of heart failure^2^. Many guidelines for the management of cardiovascular diseases refer to the importance of ventricular remodeling after AMI and its treatment modalities^3,4^. Ventricular remodeling after AMI can be divided into early ventricular remodeling and late ventricular remodeling, with various clinical manifestations, such as enlarged left ventricle, reduced left ventricular ejection fraction (LVEF), and abnormal ventricular wall motion^5,6^. Asymptomatic left ventricular systolic dysfunction (LVSD) is a major manifestation of early ventricular remodeling, with an occurrence of up to 30–60% in patients after AMI^7^. Early detection of ventricular remodeling after AMI is key to effective treatment. Although B-type brain natriuretic peptide (BNP) and echocardiography can be utilized in the diagnosis of LVSD, they carry several disadvantages, including increased cost, need for specialized technicians, and difficulty in achieving dynamic monitoring. Therefore, acoustic cardiography is gaining attention as a simple, rapid, non-invasive, and effective diagnostic modality for LVSD.

Many clinical studies have confirmed the diagnostic value of acoustic cardiography in a variety of cardiovascular diseases, especially in the diagnosis of heart failure. Some studies have shown that cardiac cycle time-corrected electromechanical activation time (EMATc), left ventricular systolic time (LVST), and third heart sound (S3) are superior to serum BNP levels in the diagnosis of heart failure. EMAT is related to left ventricular systolic function. Furthermore, S3 and S4 are less affected by age, which increase their utility in evaluating cardiac systolic and diastolic function^8–10^. However, acoustic cardiography has been rarely used in the diagnosis of coronary artery disease. Several studies have shown improved sensitivity and specificity using standard ST-T changes combined with S4 > 3.6 as diagnostic criteria for coronary artery disease^11^. Additionally, our previous study confirmed the clinical significance of acoustic cardiography in the diagnosis of coronary artery disease and ventricular remodeling^12^.

The present study investigated the relationship between parameters of acoustic cardiography and early ventricular remodeling and examined the predictive value of each parameter for early ventricular remodeling after PCI in patients with AMI.

## Methods

### Study population and study design

A total of 183 inpatients from the Departments of Cardiology of the Third Affiliated Hospital of Chongqing Medical University and Haikou People’s Hospital were enrolled in the study from March 05, 2019 to June 30, 2021 [22 patients were excluded due to poor quality electrocardiograms (ECGs)]. 161 patients with asymptomatic acute anterior wall MI after emergency PCI were included (all occurring within 24 h, with increased serum troponin I levels, ST segment elevation and pathological Q wave on ECG, and angiography showing severe stenosis or occlusion of the anterior descending branch of the coronary artery). Serum BNP, acoustic cardiography (AUDICOR, Inovise Medical, Inc., Portland, OR, USA; Henan Shanren Medical Technology Co,SR-X12Y1), and Color Doppler echocardiography were performed pre-operatively. Color Doppler echocardiography was also performed after 72 h, and LVEF% < 50% (LVEFs% were normal before operation) 72-h post-PCI was defined as early ventricular remodeling. The study subjects were divided into two groups: early ventricular remodeling group (EF% < 50% EVR, 44 cases) and normal left ventricular systolic function group (EF% ≥ 50%, Non-EVR, 117 cases).

The exclusion criteria were: (1) atrial fibrillation, pre-excitation syndrome, and intraventricular block; (2) severe liver and kidney disease; (3) patients on mechanical ventilation; (4) inferior wall MI; and (5) patients with pacemaker. Before participation, written-informed consent was obtained from each patient. We calculated the sample size through Power and Sample Size online; the website is https://powerandsamplesize.com/Calculators/.we calculated sample size between Non-EVR group and EVR group with regard to EMATc. With power equal to 0.9,we chose a ratio of 3:1 for the Non-EVR and EVR groups, the total sample size needed is 120.We have a sufficient number of cases. The study was approved by the Third Affiliated Hospital of Chongqing Medical University in the participating institutions (protocol number202133), and was conducted in accordance with the Declaration of Helsinki.

### Echocardiography

Echocardiography (Vivid 7, Vingmed-General Electric, IE33, Phillips, Andover) was performed in all patients. The biplane Simpson's method was used to calculate both end-diastolic and end-systolic volumes. These volumes were employed to calculate LVEF.

### Acoustic cardiography

**Figure 1:**
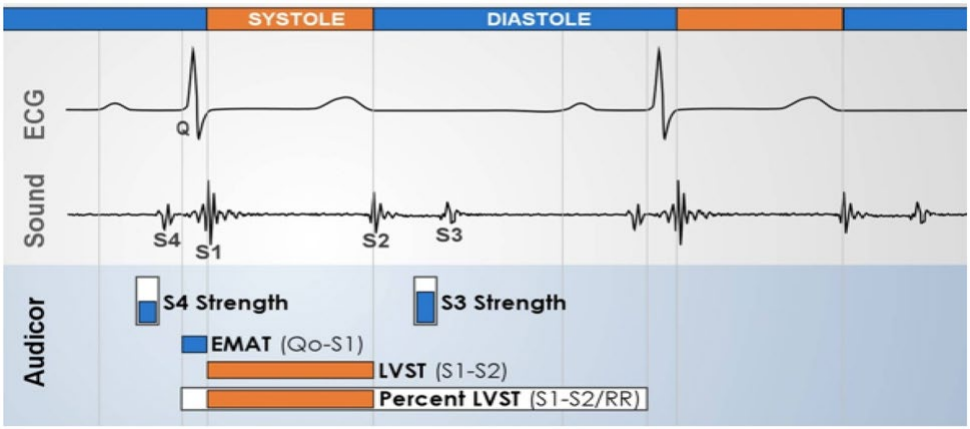
Acoustic cardiography parameters (Courtesy of Inovise Medical, Inc.). *S3* third heart sound, *S4* fourth heart sound, *EMAT* electromechanical activation time, *LVST* left ventricular systolic time.

Each patient underwent an acoustic cardiography examination in the supine position. For the measurement of heart sounds, acoustic cardiography raw data were analyzed by a computerized algorithm. At least 3 examinations were performed on each study subject, and the average values of each variable were used for analysis. In this study, the following acoustic cardiography parameters were evaluated:

1. Electromechanical activation time (EMAT), the time from Q wave onset to the mitral component of the first heart sound (S1) (Fig. 1). Time-corrected electromechanical activation time (EMATc) indicates the proportion of the cardiac cycle occupied by EMAT.
2. Fourth heart sound (S4) score: measurement of the intensity of S4 based on timing, persistency, intensity, and frequency of the sound (Fig. 1); one value between 0 and 10 is reported.
3. Third heart sound (S3) score: measurement of the intensity of S3 based on timing, persistency, intensity, and frequency of the sound (Fig. 1); one value between 0 and 10 is reported.
4. Systolic dysfunction index (SDI): SDI = exp (S3 score/10) × QRS duration × QR interval × EMAT/RR. The SDI value undergoes a nonlinear transformation and is mapped into a scale of 0–10.

### Statistical analysis

SPSS, version 20, was used for statistical analyses (SPSS, Inc., Chicago, Illinois). A two-sided *P* value < 0.05 was considered statistically significant. Continuous variables with a normal distribution were expressed as mean ± standard deviation (SD). For predicting EVR, the receiver operating characteristic (ROC) curves were generated to determine the area under the curve (AUC), sensitivity, specificity, positive likelihood ratio (LR+), and negative likelihood ratio (LR−). Multivariate logistic regression analysis was used to analyze the independent risk factors of EVR. In addition, comparisons between groups were tested by using both Student’s *t* test for normally distributed data and Mann–Whitney U test for skewed data.

## Results

### Characteristics of study subjects

A total of 161 patients were enrolled into this study. Table 1 summarizes the demographic and clinical data, medical history, and medications of our study subjects.

**Table 1:** Demographics and clinical characteristics.

Variables	Non-EVR (n = 117)	EVR (n = 44)	*P*
Age (years)	65.1 ± 7.9	64.0 ± 10.4	0.47
Male, n (%)	63 (54%)	26 (59%)	0.60
Heart rate (bpm)	79.2 ± 10.4	83.3 ± 6.1	0.02
SBP (mmHg)	141 ± 18.9	145 ± 15.44	0.20
DBP (mmHg)	77.4 ± 11.7	79.84 ± 6.9	0.19
Hypertension, n (%)	51 (43%)	17 (39%)	0.60
Diabetes, n (%)	29 (25%)	13 (29%)	0.12
ACEI/ARB, n (%)	51 (44%)	18 (41%)	0.48
Beta-blockers, n (%)	42 (36%)	14 (32%)	0.59
CCB, n (%)	43 (36%)	15 (34%)	0.47
S3	3.4 ± 0.9	3.5 ± 1.0	0.71
S4	3.7 ± 0.7	4.2 ± 1.0	0.002
EMATc	10.3 ± 1.8	13.6 ± 1.8	< 0.001
SDI	3.8 ± 0.8	4.7 ± 0.7	< 0.001
BNP(pg/ml)	82.0 ± 18.7	105.5 ± 21.9	< 0.001
LVEF	56.2 ± 4.5	41.6 ± 2.9	< 0.001

*ACEI/ARB* angiotensin converting enzyme inhibitor/angiotensin II type 1 receptor blocker, *CCB* calcium channel blocker, *SBP* systolic blood pressure, *DBP* diastolic blood pressure, *Non-EVR* acute AMI with normal ejection fraction, *EVR* acute anterior MI with ventricular remodeling.

Compared with Non-EVR patients, those EVR patients had higher EMATc, S4 score, SDI, and BNP and lower LVEF.

### Diagnostic characteristics of acoustic cardiography and BNP for detecting EVR

**Figure 2:**
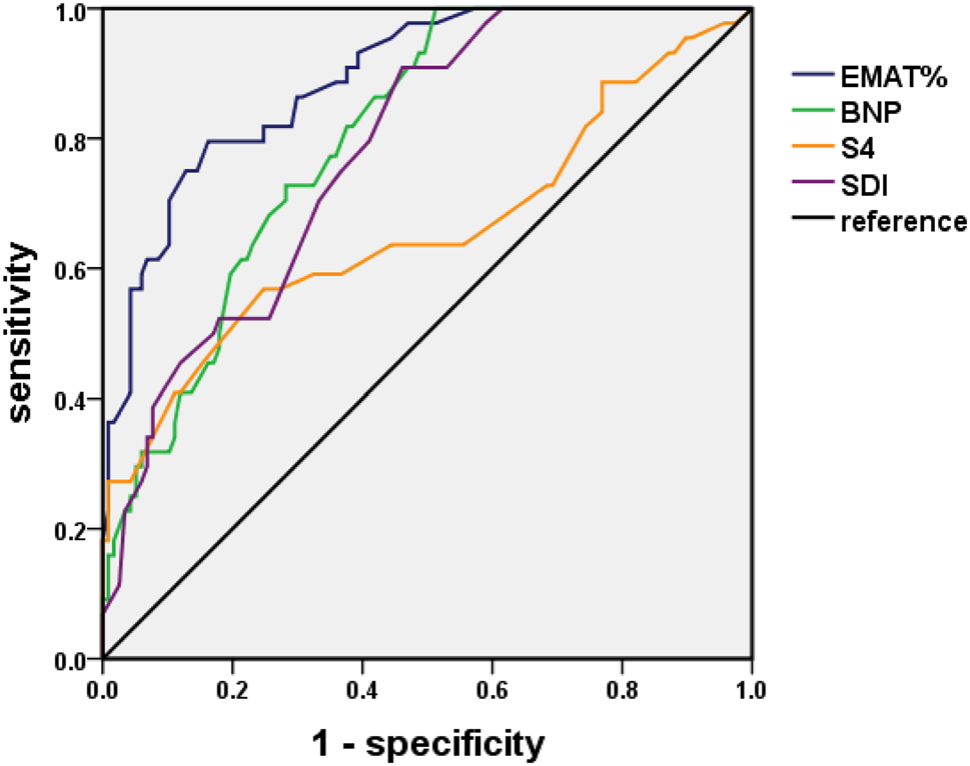
Receiver operating characteristic curves for S4 strength, EMATc, SDI, and BNP for defining EVR. The green curve represents BNP; the blue curve represents EMAT%; the yellow curve represents S4 strength; the purple curve represents SDI; EMAT% (EMATc): electromechanical activation time divided by the cardiac cycle length.

**Figure 3:**
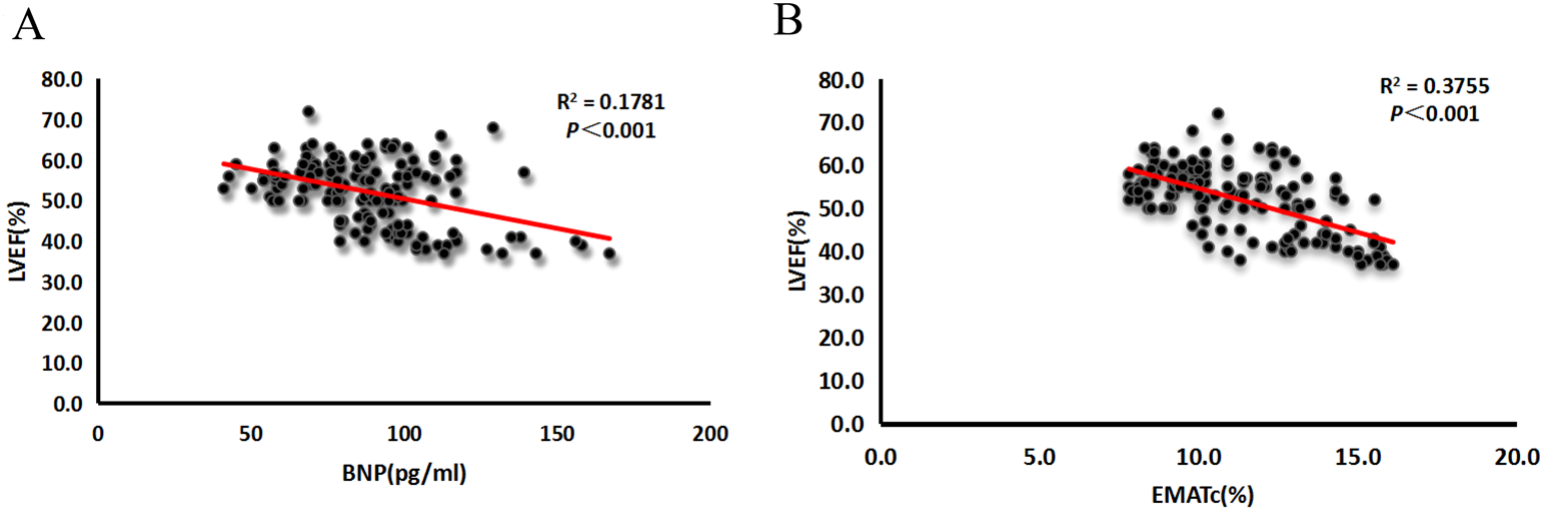
The linear correlations of LEVF, BNP and EMATc. (**A**) The linear correlations of LEVF% and BNP; (**B**) the linear correlations of LEVF% and EMATc.

By using ROC curve analyses, the value of a variety of acoustic cardiographic parameters and BNP were determined for the prediction of EVR. As shown in Fig. 2 and Table 2, the area under the ROC curve (AUC) for EMATc was 0.9 (95% confidence interval CI 0.8–0.9,* p* < 0.001). With an optimal cutoff point of 12.2, EMATc produced a sensitivity of 80% and a specificity of 83%. The AUC for S4 was 0.7 (95% CI 0.6–0.8, *p* = 0.001). With an optimal cutoff point of 4.3, S4 strength produced a sensitivity of 56% and a specificity of 75%. The AUC for SDI was 0.8 (95% CI 0.7–0.9, *p* = 0.001). With an optimal cutoff point of 3.9, SDI strength produced a sensitivity of 91% and a specificity of 54%. The AUC for BNP was 0.8 (95% CI 0.7–0.9,* p* = 0.001). In addition, with an optimal cutoff point of 92.5 pg/ml, BNP produced a sensitivity of 73% and a specificity of 72%, whereas, with an optimal cutoff point of 100 pg/ml, BNP produced a sensitivity of 46% and a specificity of 83%. As can be seen in Fig. 3, there was a better linear correlation between LVEF and EMATc than LVEF and BNP.

**Table 2:** Performance of acoustic cardiographic parameters and serum BNP levels to detect EVR.

Parameter	Cutoff value	Sensitivity	Specificity	LR+	LR−
EMATc	12.2	35/44 (80%)	20/117 (83%)	4.7	0.2
S4	4.3	19/44 (56%)	29/117 (75%)	2.2	0.6
SDI	3.9	40/44 (91%)	54/117 (54%)	2.1	0.2
BNP (pg/ml)	92.5	32/44 (73%)	33/117 (72%)	2.6	0.4
BNP (pg/ml)	100	20/44 (46%)	97/117 (83%)	2.8	0.7

### Determination of independent risk factors for EVR

As shown in Table 3, many parameters (SDI, HR, S4, S3, BNP, age, sex, diabetes, and EMATc) were analyzed for statistically significant differences by multivariate logistic regression. The parameters, EMATc (*OR* 2.3, 95% *CI* 1.4–3.7, *p* < 0.001), SDI (*OR* 15.9, 95% *CI* 3.6–69.5, *p* = 0.001), and BNP (*OR* 1.1, 95% *CI* 1.0–1.1, *p* = 0.009), were independent risk factors for EVR. There were no statistical differences in the parameters of S3, S4, HR, sex, diabetes, and age.

**Table 3:** Determination of independent risk factors for EVR.

Variables	OR	95% CI	*P*
S3	1.2	0.5–2.9	0.67
S4	2.4	0.8–7.4	0.13
EMATc	2.3	1.4–3.7	< 0.001
SDI	15.9	3.6–69.5	0.001
BNP	1.1	1.0–1.1	0.009
HR	1.4	0.9–2.0	0.23
Age	0.9	0.9–1.1	0.53
Sex	2.5	0.5–13.4	0.29
Diabetes	0.4	0.07–2.0	0.24

## Discussion

Although the widespread use of PCI has improved the prognosis of patients with AMI, many patients still develop heart failure. Ventricular remodeling after AMI is an independent predictor of the development of HF and is one of the major factors affecting prognosis but without uniform and clear criteria^1^. It has suggested that > 15% increase in left ventricular end systolic volume, measured by echocardiography, 6 months after PCI for AMI, is evidence of ventricular remodeling^13^. However, a meta-analysis of data from Medline and Embase showed that the LVEF was below 50% in patients with asymptomatic LVSD after AMI^14^. In a prospective study of 284 patients with AMI treated with PCI, 30% had ventricular remodeling after 6 months^15^. Early ventricular remodeling usually occurs 24–72 h after AMI and presents clinically as asymptomatic LVSD, but is underdiagnosed^5,7^. There are a number of clinical indicators that predict LVSD;copeptin was shown to be a valid indicator^16^;heart rate-corrected QT interval prolongation to the Global Registry of Acute Coronary Events risk score improves the predictive value for early mortality in patients with acute coronary syndrome^17^; Of course, there are many other methods of prediction, such as BNP, echocardiography, and magnetic resonance imaging,, but these approaches are expensive, cumbersome and cannot be monitored systematically. Moreover, many patients refused these tests to be performed. Therefore, acoustic cardiography is gaining attention as a simple, rapid, non-invasive, and effective diagnostic modality for asymptomatic LVSD which leads to HF. The practical value of acoustic cardiography, with EMATc, as the main parameter, in the clinical diagnosis of heart failure has also been reported. Dillier et al.^10^ showed that in healthy subjects there was no circadian rhythm variation in EMAT, no significant change from wake-to-sleep state, and no change with age. However, prolonged EMATc in patients with HF is associated with LVSD, poor HF prognosis, and rehospitalization, independent of serum BNP levels and LVEF^9,18^. Roos et al.^19^ reported that in 108 patients who underwent elective diagnostic cardiac catheterization, EMAT > 110 ms had a high specificity for LVEF (~ 50%) patients with LVSD. In a study of 433 patients, Kosmicki et al.^8^ found that EMAT, LVST, and S3 were better than serum BNP in the diagnosis of LVSD; acoustic cardiography alone and acoustic cardiography combined with BNP had equivalent clinical value in the diagnosis of LVSD; in the "gray zone" of serum BNP (100 ng/L to ≤ 500 ng/L), acoustic cardiography was superior to BNP. Many other studies have found the clinical applications of EMATc in disease prediction. Zhang et al. reported that the elevated EMATc measured on admission was an independent risk factor for major adverse cardiovascular events in hospitalized patients with congestive heart failure. Acoustic cardiography measured on admission may provide a simple, non-invasive method for risk stratification of patients with congestive heart failure^20^. Chao et al. found that EMAT-guided post-discharge management was superior to traditional symptom-driven therapy in patients hospitalized for acute heart failure in terms of 1-year outcomes^18^. There are few clinical studies on the value of acoustic cardiography in the diagnosis of AMI. Some studies have shown that ST­T changes combined with S4 as the standard for the diagnosis of coronary heart disease have a sensitivity and specificity of 68% and 84% respectively; S3 or S4 combined with ECG increases the detection rate of myocardial ischemia by 32%^11,21^.

In the present study, we identified three parameters, EMATc, S4, and SDI, as independent risk factors for post-PCI early ventricular remodeling in patients with AMI and their predictive value for the occurrence of early ventricular remodeling. We determined the value of various acoustic cardiographic parameters and serum BNP levels for predicting early ventricular remodeling and showed that the AUC of EMATc was 0.89, the optimal threshold was 12.2, the sensitivity of EMATc was 80%, and the specificity was 83%. By contrast, when the optimal threshold of serum BNP was set at 100 pg/ml, the sensitivity of BNP was 46% and the specificity was 83%. Therefore, our results suggested that EMATc was superior to serum BNP levels.

We believe that acoustic cardiography is a simple, quick, and effective way to diagnose early ventricular remodeling after AMI. However, there are some limitations of the present study that need to be addressed. First, the sample size was small (n = 161). Therefore, more subjects are needed to determine the clinical value of acoustic cardiography in the diagnosis of AMI. Second, we selected only the patients with anterior MI. Third, echocardiography data were limited to EF% values and therefore, did not provide additional information on other echocardiography variables that may have led to the discovery of other significant correlations. A better way to determine early ventricular remodeling should be the decrease in LVEF after PCI compared with pre-PCI LVEF. However, echocardiography is often overlooked in order to complete the procedure as quickly as possible pre-PCI. Therefore, it was difficult for us to obtain complete information. Finally, we only studied the early ventricular remodeling rather than the relationship between acoustic cardiography and late ventricular remodeling, which would take a long time.

## Conclusions

We identified three parameters, EMATc, S4, and SDI, as independent risk factors for post-PCI early ventricular remodeling in patients with AMI and also identified the predictive value of these parameters for the occurrence of early ventricular remodeling in these patients. Acoustic cardiography can be a simple, quick, and effective way to diagnose early ventricular remodeling after AMI.

## Data Availability

The datasets generated and analyzed in the present study are available from the corresponding author upon reasonable request.
